# Clinical Utility of microRNAs in Exhaled Breath Condensate as Biomarkers for Lung Cancer

**DOI:** 10.3390/jpm11020111

**Published:** 2021-02-09

**Authors:** Carlos Pérez-Sánchez, Nuria Barbarroja, Lucas C. Pantaleão, Laura M. López-Sánchez, Susan E. Ozanne, Bernabé Jurado-Gámez, Enrique Aranda, Chary Lopez-Pedrera, Antonio Rodríguez-Ariza

**Affiliations:** 1 Department of Medicine, School of Clinical Medicine, Cambridge Biomedical Campus, University of Cambridge, Cambridge CB2 0AW, UK; 2 Maimonides Institute for Biomedical Research of Cordoba (IMIBIC), Reina Sofia University Hospital, University of Cordoba, 14004 Córdoba, Spain; lmlopezsanchez@gmail.com (L.M.L.-S.); bjg01co@hotmail.com (B.J.-G.); earandaa@seom.org (E.A.); charylopezpedrera@gmail.com (C.L.-P.); antonio.rodriguez.exts@juntadeandalucia.es (A.R.-A.); 3 Metabolic Research Laboratories and MRC Metabolic Diseases Unit, Welcome Trust-MRC Institute of Metabolic Science, Addenbrooke’s Hospital, University of Cambridge, Cambridge CB2 0QQ, UK; lp435@medschl.cam.ac.uk (L.C.P.); seo10@medschl.cam.ac.uk (S.E.O.); 4 Centro de Investigación Biomédica en Red en Cáncer (CIBERONC), 28029 Madrid, Spain

**Keywords:** exhaled breath condensate, microRNA, lung cancer, biomarkers, personalized medicine

## Abstract

This study represents a novel proof of concept of the clinical utility of miRNAs from exhaled breath condensate (EBC) as biomarkers of lung cancer (LC). Genome-wide miRNA profiling and machine learning analysis were performed on EBC from 21 healthy volunteers and 21 LC patients. The levels of 12 miRNAs were significantly altered in EBC from LC patients where a specific signature of miR-4507, miR-6777-5p and miR-451a distinguished these patients with high accuracy. Besides, a distinctive miRNA profile between LC adenocarcinoma and squamous cell carcinoma was observed, where a combined panel of miR-4529-3p, miR-8075 and miR-7704 enabling discrimination between them. EBC levels of miR-6777-5p, 6780a-5p and miR-877-5p predicted clinical outcome at 500 days. Two additional miRNA signatures were also associated with other clinical features such as stage and invasion status. Dysregulated EBC miRNAs showed potential target genes related to LC pathogenesis, including CDKN2B, PTEN, TP53, BCL2, KRAS and EGFR. We conclude that EBC miRNAs might allow the identification, stratification and monitorization of LC, which could lead to the development of precision medicine in this and other respiratory diseases.

## 1. Introduction

Lung cancer is the most commonly diagnosed cancer and the leading cause of cancer death worldwide, with more than 1.8 million deaths in 2018, according to a GLOBOCAN 2018 estimate [1]. The average 5-year survival rate of lung cancer is disappointingly low with a mortality rate of 85%. Most patients are diagnosed at an advanced stage, and half of them will die within 1 year of diagnosis since at the time of diagnosis, only 15% of tumors are still localized allowing surgical resection. The most common type of lung cancer is non-small cell lung cancer (NSCLC), accounting for nearly 90% of all cases. The two main types of NSLC are adenocarcinoma (AD) and squamous cell carcinoma (SCC, also known as epidermoid carcinoma), which account for 30% and 25% of all lung cancers, respectively [2]. Although there have been recent advances in novel therapies for lung cancer, they are not effective at an advanced stage of the disease and, therefore, these treatments have not improved the prognosis for those patients [3]. Thus, an effective early screening test has been long sought to identify the disease in early stages, when treatments are much more effective. Chest radiography and sputum cytology have been tested in early screening trials, however, they were not effective in reducing lung cancer mortality rates [4]. More recently, screening with low-dose computed tomography (CT) has also been tested in several clinical trials, but data from these studies are controversial since some studies showed a reduction in mortality versus screening with standard chest X-rays [5,6] while other smaller randomized controlled trials were inconclusive [7,8,9]. Apart from the fact that false-positive results have been commonly detected in most of these screening trials, it is also questionable whether low-dose CT could be translated into clinical practice because of the resources and expertise required for the acquisition and interpretation of images. Thus, it is crucial to develop accessible diagnostic tools to detect disease early, as well as the type, the stage and the invasion capability of the tumor. We previously identified sensitive biomarkers of lung adenocarcinoma through proteomic analysis of bronchoalveolar lavage fluid [10]. However, this requires invasive sampling so it is not suitable for the screening and early diagnosis of lung cancer. In contrast, exhaled breath condensate (EBC) contains material from the lungs and the lower respiratory tract and can be collected non-invasively. Therefore, it could be very useful in the diagnosis and prognosis of lung cancer [11]. There are several methods that can be applied to analyze the EBC for the identification of volatile compounds emitted by tumor growth, including spectrographic techniques, e-nose and trained dogs [12]. Thus, Philip et al. found a specific alteration of some volatile organic compounds (VOCs) present in EBC that could be considered as a screen for lung cancer in adult smokers [13]. Moreover, the combination of VOCs and chest CT even potentially could improve the sensitivity and specificity of lung cancer diagnosis [14].

A number of other molecules have been reported to be present in EBC, including polypeptides, proteins, and nucleic acids (DNA, mitochondrial DNA and microRNAs) [15]. In addition, mRNA isoforms of GATA6 (GATA-binding factor 6) and NKK2-1 (NK2 homeobox 1) in EBC have been proposed for lung cancer detection [16]. In this scenario, we have recently reported that the proteomic analysis of EBC samples is an appropriate approach to develop biomarkers for the diagnosis of lung cancer [17]. MicroRNAs (miRNAs) are small RNAs that by complementary sequence regulate gene expression promoting the degradation of their mRNA target genes or repressing protein translation. MiRNAs are key players in multiple cellular processes and their alteration has been associated with different diseases such as cancer [18]. The marked stability of cell-free miRNAs in body fluids and the possibility of the detection of a small number of copies represent an advantage of using miRNAs as biomarkers over other markers. Additionally, miRNAs levels in the circulation change under pathological conditions as they can be passively and actively secreted from diseased tissues. That detectable changes make cell-free miRNAs promising non-invasive biomarkers for diagnosis and disease progression.

The utility of miRNAs as biomarkers in respiratory diseases has been widely reported [19], however, their potential use as clinical tools in non-invasive samples such as EBC has not yet been fully explored.

**Figure 1:**
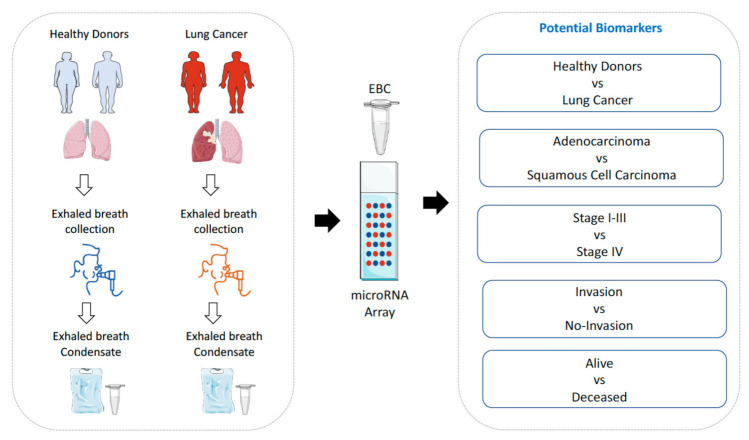
Overview of the study design. Genome-wide miRNA expression profiling was performed on exhaled breath condensate (EBC) from 21 healthy volunteers and 21 lung cancer patients. The utility of EBC miRNAs as biomarkers of diagnosis, type of tumor, stage, invasion capacity and prognosis was evaluated.

Here we report for the first time the genome-wide miRNA expression profile in EBC samples from lung cancer patients as proof of concept of their potential clinical utility in the diagnosis, prognosis and management of respiratory diseases (study design is displayed in Figure 1).

## 2. Materials and Methods

### 2.1. Subjects

This is a cross-sectional study including 42 subjects from the Respiratory Medicine Department of the Reina Sofia Hospital (Córdoba, Spain).

The research was performed following the ethics code of the World Medical Association (Declaration of Helsinki). All participants signed the informed written consent. The approval of the Ethics Committee of the Reina Sofia Hospital was obtained. The EBC samples from 21 healthy donors and 21 patients diagnosed with lung cancer were collected under fasting conditions and stored at −80 °C.

Lung cancer patients included 19 males and 2 females with an age of 62 ± 8 years, while healthy donors were integrated by 20 males, 1 female with an age of 61 ± 9 years. The type of lung cancer present in our cohort was AD (57%) and SCC (43%). Invasion was present in 53% of lung cancer patients, while 60% showed metastatic cancer. Stage IV was the most prevalent with 57%, following by stage III, II and I with 24, 14 and 5% respectively, and the tumor size was of 49 ± 23 cm. Clinical details of the lung cancer patients and healthy donors are displayed in Table 1.

The diagnosis included clinical tests performed on the 21 patients, based on fine-needle biopsy, bronchoscopy, video-assisted thoracoscopy and subsequent cytohistology confirmation. Subjects with coexistence of severe extrapulmonary disorder (grade IV cardiac insufficiency according to the New York Heart Association, advanced hepatic cirrhosis, stage V renal insufficiency), extrapulmonary neoplasm in the last five years, specific lung disease not related to smoking (including pneumonia, interstitial pneumopathy, tuberculosis, etc.) and unjustified weight loss in the last year were excluded from both groups.

### 2.2. EBC Collection

By using the EcoScreen 2 device (FILT Lungen-und Thoraxdiagnostik, Berlin, Germany) EBC was collected. Subjects wearing a nose clip breathed at a normal frequency and continuous volume for a 15-min period through a disposable mouthpiece. The system contained different valves that allowed the segregation of inhaled air from exhaled air. Thereafter, the breath was driven to a cooling device, which condensed and separated the EBC into two disposable polyethylene bags containing the EBC from the upper and the distal airways. This approach avoids saliva contamination. Besides, a protection filter (Scharlab, Barcelona, Spain) located over the inlet air valve does not allow the exogenous particles enter from the atmosphere of the room.

In this study, EBC from lower airways was used in order to reduce the contribution of upper airways (environmental factors influence) and increase the bronchial and alveolar tracts involvement.

### 2.3. RNA Isolation

RNA was isolated from lower respiratory tract EBC by using the miRNeasy Serum/Plasma Kit (Qiagen, Hilden, Germany). This kit purifies cell-free total RNA, which primarily includes small RNAs such as miRNAs, from small volumes from diverse body fluids. In brief, two hundred microliters of EBC was mixed with denaturing buffer. Next, the manufacturer’s protocols were followed for RNA extraction. Finally, RNA was recovered in 14 μL of RNase-free water. The RNA concentration was quantified by NanoDrop ND-1000 (Nanodrop Technologies LLC, Wilmington, DE, USA).

### 2.4. Genome-Wide miRNA Expression Profiling

MicroRNA expression profile was carried out through GeneChipR miRNA 4.0 Array using Affymetrix technology (Affymetrix, Sunnyvale, Santa Clara, CA, USA). The platform contained 30424 probe sets designed to detect all mature human miRNA sequences (2578) included in miRBase Release 20. Microarray experiments were conducted in all samples following the manufacturer’s recommendations. Shortly, 5ul of total RNA was labeled with FlashTag Biotin Labeling Kit and hybridized in the Affymetrix Hybridization Oven 640 at 48 °C overnight. Using fluidics script FS450_0003 the arrays were stained and then scanned on an Axon 4000B microarray scanner (Axon Instruments, Foster City, CA, USA).

Raw data were processed using oligo package for R [20]. Expression data was obtained by RNA background subtraction (treating the intensities of probe signals as a convolution of noise and true signals), quantile normalization and median-polish summarization of raw multichip data. Quality control of the output data was assessed using tests embedded into the oligo package.

### 2.5. Data Analysis and Statistics

Prior to differential gene expression analysis, probes not designed for human miRNA detection were trimmed out from the dataset. We then selected the 5% more abundant human miRNAs according to the individual median calculated across datasets and carried out least squares regression statistical analysis available in the limma package for R to identify differential gene expression between two groups [21]. Differences in miRNA expression were deemed significant when *p* < 0.05. For each group comparison in this study, we also performed a principal component analysis of scaled data from regulated microRNAs. Scaled data was obtained by subtracting individual miRNA levels average from the corresponding miRNA levels of individuals samples, and by dividing the now centered results by their standard deviations. We also designed training classification models to detect variable importance amongst regulated miRNAs. A random forest algorithm was therefore applied repeatedly. At each iteration, the number of variables randomly sampled as candidates at each split in the decision trees was determined by testing the accuracy of cross-validated random forests (500 trees), and the final model was designed using the number of variables at each split that returned the best accuracy. Individual miRNA importance in the model at each interaction, indicating the mean decrease in accuracy by rearranging the values of each variable, was then identified and recorded. The least important variable was identified and removed from the next iteration until the 3 miRNAs with the highest importance were identified. We then used random forest models with leave-one-out cross-validation (LOOCV) for the seven grouping possibilities to identify the most accurate combination, and a binomial generalized linear model fitted with the selected miRNAs was generated. Finally, we determined the sensitivity and specificity of the individual selected miRNAs and the fitted model at various thresholds to generate ROC curves. We then calculated the area under the curve to determine the diagnostic ability of individual miRNAs and the fitted model to predict the outcome.

### 2.6. Target Gene Prediction and Integrated Analysis by IPA

The panel of EBC miRNAs associated with lung cancer patients and their clinical characteristics were analysed to gain insight into the biological pathways, functions and networks by using the Ingenuity Pathway Software (IPA). Potential mRNA targets (predicted with high confidence and experimentally observed) of the EBC-miRNA signature were recognised through different predicted tools (TargetScan, miRecords, TarBase, and Ingenuity^®^ knowledge Base) (IPA, Quiagen). Through the microRNA target filter tool, we selected only mRNA targets showing either experimentally observed or high confidence predicted interactions, which have been previously associated with the pathogenesis of lung cancer.

## 3. Results

### 3.1. Lung Cancer Patients Exhibit an Altered EBC miRNA Profile

**Figure 2:**
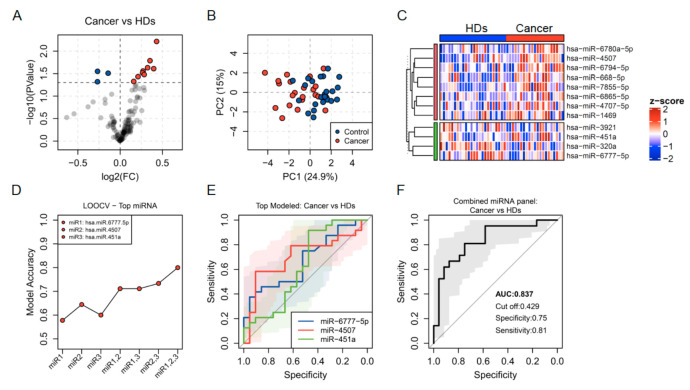
(**A**) Volcano plot showing fold change (FC) and statistical significance of individual miRNA levels in the breath condensate of cancer patients compared to healthy donors. Blue and red dots represent negatively and positively regulated miRNAs respectively (*p* < 0.05). (**B**) Regulated miRNAs calculated PCA plots showing the PC1 and PC2 scores for individual cancer patients (red dots) and healthy donors (blue dots). (**C**) Heatmap showing z-score of miRNAs regulated in healthy donors and cancer patients. (**D**) Accuracy of lung cancer prediction calculated by LOOCV models for individual miRNAs and for the four possible combinations. (**E**,**F**) ROC curves predicted for an individual (**E**) and combined miRNAs (**F**) selected through a random forest iteration model.

Using GeneChipR miRNA 4.0 array we profiled the miRNA expression of ECB samples from 42 subjects (21 lung cancer patients and 21 healthy controls). By applying a robust multichip average algorithm, used to subtract background, normalise quantiles and summarise raw microarray fluorescence data, the expression data from the 42 subjects included in the study was obtained. Using a multiple-linear-model approach, we identified a set of individual transcripts altered in lung cancer (*p*-value < 0.05) amongst the top 5% most abundant miRNAs. Of these, 128 miRNAs, 9 miRNAs were significantly upregulated (miR-6865-5p, miR-4707-5p, miR-451a, miR-1469, miR-4507, miR-6780a-5p, miR-668-5p, miR-6794-5p and miR-7855-5p) while 3 were downregulated (miR-3921, miR-320a, miR-6777-5p) in breath condensates from lung cancer patients (Figure 2A).

A principal component analysis of scaled results from the differentially expressed miRNAs showed a separation between samples from cancer patients and healthy donors (HDs) in component 1, which explains 24.9% of the variance observed (Figure 2B). A heat map including the levels and clustering of those individual transcripts was also obtained (Figure 2C).

To identify the most suitable miRNAs in the breath condensate to be used as biomarkers of lung cancer, we designed training classification random forest models (500 trees) to detect variable importance. The algorithm was applied repeatedly, and at each iteration, the least important variable was flagged and removed from the next iteration until the 3 miRNAs with the highest importance were identified. We then developed random forest models with leave-one-out cross-validation (LOOCV) for the seven grouping possibilities using the selected variables and identified the model using all three miRNAs as the most accurate one (Figure 2D).

At the individual level, miR-4507 showed the best specificity and sensitivity, with the largest area under the curve (AUC of 0.70) in a receiver operating characteristic (ROC) plot followed by miR-6777-5p and miR-451a (AUC of 0.66 and 0.63, respectively) (Figure 2E). A binomial generalised linear model fitted with the three selected miRNAs was more robust and showed a higher AUC of 0.83 (Figure 2F).

### 3.2. EBC miRNAs Discriminate between the Two Most Common Types of Lung Cancer AD and SCC with High Accuracy

**Figure 3:**
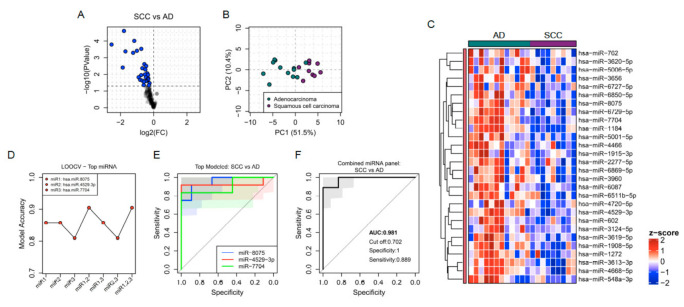
(**A**) Volcano plot showing fold change (FC) and statistical significance of individual miRNA levels in the breath condensate of SCC (squamous cell carcinoma) compared to AD (adenocarcinoma) patients. Blue dots represent negatively regulated miRNAs respectively (*p* < 0.05). (**B**) Regulated miRNAs calculated PCA plots showing the PC1 and PC2 scores for individual SCC patients (purple dots) and AD patients (green dots). (**C**) Heatmap showing z-score of miRNAs regulated in EBC from SCC and AD patients. (**D**) Accuracy of specific cancer type prediction calculated by LOOCV models for individual miRNAs and for the four possible combinations. (**E**,**F**) ROC curves predicted for an individual (**E**) and combined miRNAs (**F**) selected through a random forest iteration model.

Twenty-eight miRNAs were differentially expressed in EBC from AD compared to SCC lung cancer patients (Figure 3A). The PCA analysis of those miRNAs might distinguish patients from both types of cancer (Figure 3B). The expression level of the signature consisting of 28 miRNAs was significantly reduced in SCC compared with AD patients (Figure 3C). The top three biomarker miRNAs able to distinguish AD from SCC lung cancer with the highest accuracy were miR-4529-3p, miR-8075 and miR-7704 after LOOCV and ROC curve analysis (Figure 3D,E). The combined panel integrated by those three miRNAs improved the individual capacity to discriminate between both types of lung cancer with an AUC of 0.98, 100% specificity and 88% sensitivity (Figure 3F).

### 3.3. Association of EBC miRNAs with Clinical Features of Lung Cancer: Stage and Invasion

**Figure 4:**
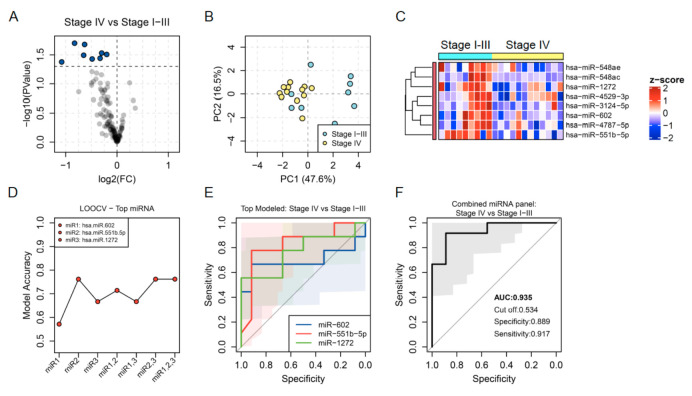
(**A**) Volcano plot showing fold change (FC) and statistical significance of individual miRNA levels in the breath condensate of lung cancer patients with stage IV compared to stage I-III. Blue dots represent negatively regulated miRNAs (*p* < 0.05). (**B**) Regulated miRNAs calculated PCA plots showing the PC1 and PC2 scores for individual lung cancer patients with stage IV (yellow dots) and stage I-III (blue dots). (**C**) Heatmap showing z-score of miRNAs regulated in EBC from lung cancer patients with stage I-III and stage IV. (**D**) Accuracy of stage prediction calculated by LOOCV models for individual miRNAs and for the four possible combinations. (**E**,**F**) ROC curves predicted for an individual (**E**) and combined miRNAs (**F**) selected through a random forest iteration model.

Three specific signatures of EBC miRNAs distinguished clinical characteristics of lung cancer including stage and invasion capability. A signature of 8 EBC miRNAs was downregulated in patients with stage IV compared to those patients with less advanced disease defined by stages I, II or III. This EBC miRNA signature was composed of miR-548ae, miR-548ac, miR-1272, miR-4529-3p, miR-3124-5p, miR-602, miR-4787-5p and miR-551b-5p (Figure 4A–C). The best EBC miRNA candidates as biomarkers to identify patients with the most advanced stage of the disease were miR-602, miR-551b-5p and miR-1272 and the combined panel of these three miRNAs improved the discrimination capacity of each miRNA independently with an AUC of 0.88, 89% specificity and 92% sensitivity (Figure 4D–F).

**Figure 5:**
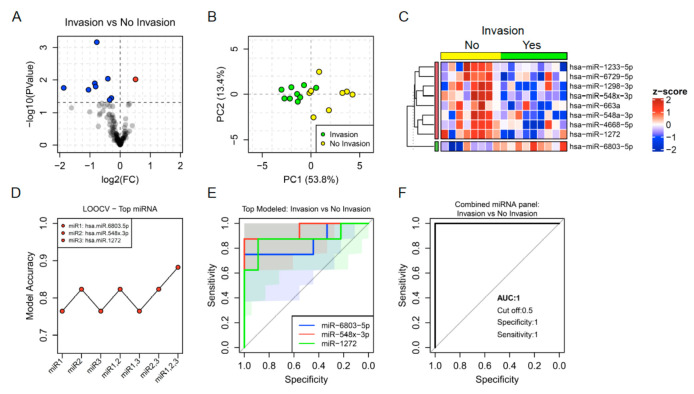
(**A**) Volcano plot showing fold change (FC) and statistical significance of individual miRNA levels in the breath condensate of lung cancer patients with invasion compared to those having no invasion. Blue and red dots represent negatively and positively regulated miRNAs respectively (*p* < 0.05). (**B**) Regulated miRNAs calculated PCA plots showing the PC1 and PC2 scores for individual lung cancer patients with invasion (green dots) and patients having no invasion (yellow dots). (**C**) Heatmap showing z-score of miRNAs regulated in EBC from lung cancer patients with and without invasion. (**D**) Accuracy of invasion capacity prediction calculated by LOOCV models for individual miRNAs and for the four possible combinations. (**E**,**F**) ROC curves predicted for an individual (**E**) and combined miRNAs (**F**) selected through a random forest iteration model.

Regarding the invasion capability of the tumors, a signature of 9 EBC miRNAs was differentially expressed between invasive and non-invasive tumors. Invasive tumors were characterised by the downregulation of miR-1233-5p, miR-6729-5p, miR-1298-3p, miR-548x-3p, miR-548a-3p, miR-4668-5p, miR-663a, miR-1272, and the upregulation of miR-6803-5p (Figure 5A–C). Three of these EBC miRNAs showed the highest accuracy as biomarkers of invasion in lung cancer tumors including miR-6803-5p, miR-548x-3p and miR-1272. The combination of these miRNAs in an integrated panel distinguished invasive from non-invasive tumors with 100% specificity and sensitivity in our cohort (Figure 5D–F).

### 3.4. EBC miRNAs Predict the Clinical Outcome of Lung Cancer Patients with High Accuracy

In order to evaluate the capacity of EBC miRNAs as biomarkers of prognosis, we performed a prospective clinical follow-up of all the patients included in this study for 500 days since the first EBC sample was collected. The final clinical outcome of lung cancer patients after this time was defined as deceased or alive. Ten lung cancer patients were deceased and 8 were alive after 500 days. At this point, data from 3 patients were missing. Among the deceased patients, 80% were at stage IV and 20% were at stage I-III at the moment of EBC sample collection. In contrast, 25% of the alive patients were at stage IV and 75% were at stage I-III by the time of EBC testing.

**Figure 6:**
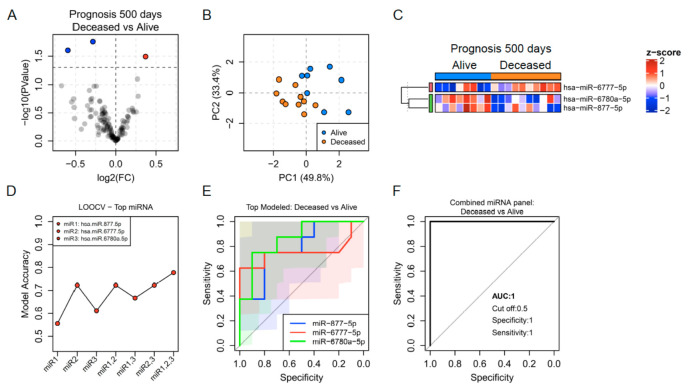
(**A**) Volcano plot showing fold change (FC) and statistical significance of individual miRNA levels in the breath condensate of lung cancer patients who were deceased after 500 days and those alive. Blue and red dots represent negatively and positively regulated miRNAs respectively (*p* < 0.05). (**B**) Regulated miRNAs calculated PCA plots showing the PC1 and PC2 scores for individual lung cancer patients alive (blue dots) and deceased (orange dots). (**C**) Heatmap showing z-score of miRNAs regulated in EBC from lung cancer patients who died and are alive after 500 days. (**D**) Accuracy of invasion capacity prediction calculated by LOOCV models for individual miRNAs and for the four possible combinations. (**E**,**F**) ROC curves predicted for an individual (**E**) and combined miRNAs (**F**) selected through a random forest iteration model.

A signature of three miRNAs was differentially expressed at baseline between the two prognosis groups. The expression level of miR-6777-5p was up-regulated, while 6780a-5p and miR-877-5p were downregulated in the deceased group compared with the alive group after 500 days. A principal component analysis highlighted the stratification of both prognosis groups based on the expression of these miRNAs (Figure 6A–C). The potential of the EBC miRNA signature as biomarker predictors of prognosis in lung cancer patients was demonstrated through the ROC curve analysis which showed individual AUCs above 0.8. The combined model integrated by those three miRNAs predicted the prognosis at 500 days with 100% specificity and sensitivity along with an AUC of 1 in our cohort of patients (Figure 6D–F). Interestingly, as 20–25% of lung cancer patients did not show a correlation between their stage and outcome at 500 days, the panel of miRNAs predictors of each clinical feature was independent.

Taken together, our results highlight the potential of several miRNAs as useful biomarkers candidates for diagnosis, prognosis and clinical features of lung cancer (Table 2).

### 3.5. The EBC miRNA Signature Associated with Lung Cancer and Its Clinical Features Shows Multiple Potential mRNA Targets Related to the Disease

**Figure 7:**
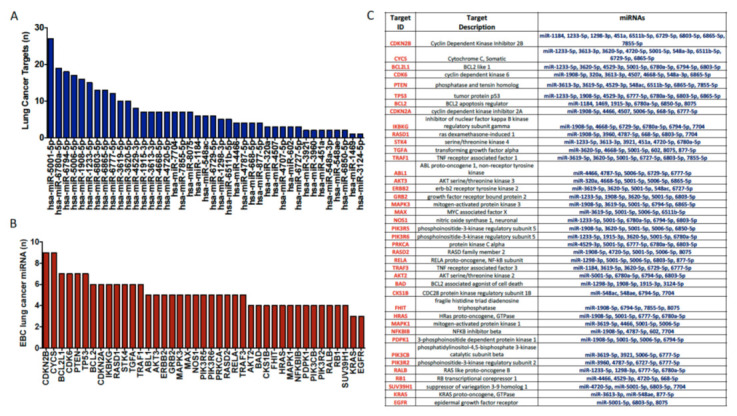
(**A**) Number of potential mRNA targets of the miRNAs identified in EBC that were signatures of lung cancer and associated with its clinical features. Individual miRNAs can simultaneously interact with multiples mRNA targets. (**B**,**C**) A number of individual lung cancer-associated mRNA targets could be simultaneously regulated by multiples EBC miRNAs.

Ingenuity Pathway Analysis allowed us to identify potential targets of the miRNAs identified in EBC that were signatures of lung cancer and associated with its clinical features. This included a remarkable number of potential targets known to be related to the pathogenesis of the disease. Thus, a complex network between EBC miRNAs and lung cancer-associated mRNA targets was obtained where individual miRNAs simultaneously interact with multiples mRNA targets. Among these miRNAs, miR-5001-5p, -6780a-5p, -6794-5p, -5006-5p, -1908-5p, -1233-5p, -6803-5p, -6865-5p, and miR-6777-5p showed more than 10 potential lung cancer-associated mRNA targets at the same time (Figure 7A). Reciprocally, in this network, a number of individual lung cancer-associated mRNA targets could be simultaneously regulated by multiples EBC miRNAs. Among these lung cancer-associated genes, CDKN2B, CYCS, BCL2L1, CDK6, PTEN, TP53, BCL2, CDKN2A, IKBKG, RASD1, STK4, TGFA and TRAF1, were predicted to be regulated by more than 5 EBC miRNAs at the same time (Figure 7B,C).

## 4. Discussion

We here show the proof of concept of the clinical utility of miRNAs from EBC as non-invasive biomarkers for lung cancer. Previous studies have shown the potential of EBC miRNAs as biomarkers for other respiratory diseases such as asthma, Chronic Obstructive Pulmonary Disease (COPD), and pulmonary tuberculosis [22,23,24]. Pinkerton et al. analysed a candidate panel of 39 miRNAs by PCR in 11 patients with asthma, 10 with COPD and 12 healthy volunteers (HDs). Three miRNAs including miR-1248, miR-1291 and let-7 were downregulated in asthma compared with COPD and HDs, while miR-328 and miR-21 were lower in both respiratory diseases compared with HDs [22]. Sinha et al. also analysed a larger candidate panel of miRNAs in EBC from 10 patients with asthma and 10 HDs by PCR. Seven miRNAs (miR-649, 1264, 2861, 574-5p, 453, 4256, 556-5p) were simultaneously altered in both asthma and pulmonary tuberculosis, although to a much higher degree in the tuberculosis group. They demonstrated that most of the detected miRNAs in EBC were present in exosomes allowing the transference of miRNAs between cells and increasing their stability in this biofluid [23]. To date, the only previous study of EBC in relation to lung cancer, was by Monzzoni et al. who analysed the levels of two selected miRNAs in EBC and plasma from 54 NSCLC and 46 HDs by PCR. They showed upregulation of miR-21 and downregulation of miR-486 in both EBC and plasma from lung cancer patients, suggesting the clinical utility of miRNAs as biomarkers for the diagnosis of NSCLC [24].

Our data showed that the unsupervised analysis of the whole miRNome in EBC allows the identification of miRNA signatures that might differentiate individuals with lung cancer. New studies with large cohorts of patients are needed to replicate and validate these and other models that would significantly improve the early detection and management of this and other respiratory diseases.

Currently, the only way to diagnose the type of lung cancer is through invasive biopsy techniques. This is a critical step for the clinical decision of selecting the most suitable therapy for each patient. Several studies have identified potential miRNAs candidates as biomarkers for typifying the type of cancer in histological samples. For example, Zhang et al. identified four miRNAs highly expressed in SCC including miR-93, miR-205, miR-221 and miR-30e, while adenocarcinoma was characterised by high expression of five miRNAs like miR-29b, let-7e, miR-100, miR-29c and miR-125a-5p [25]. The specificity of miR-205 as biomarker of SCC in lung biopsies has been confirmed in other two additional studies [26,27]. In adenocarcinoma, Nadal et al. showed that miR-411, miR-370 and miR-376a, were associated with poor survival after resection [28]. The translational potential of these histological biomarkers was also demonstrated by the development of a microRNA-based diagnostic assay called “miRview lung”, which distinguished four main lung cancer subtypes with high accuracy (94%) using the expression of a signature of 8 miRNAs (miR-205, miR-21, miR-125a-5p, miR-29b, miR-106a, miR-129-3p, miR-7, and miR-375) [29]. In our study, we showed for the first time, that miRNAs coming directly from the respiratory tract in non-invasive samples such as EBC can also identify the type of lung cancer with a high degree of accuracy in a small cohort of patients. These promising results open the door for new clinical studies to confirm the clinical utility of this non-invasive test which might influence the early diagnosis, treatment and quality of life of these patients.

Despite the standard therapies, the severity and rapid progression of lung cancer are associated with the aggressive behaviour of the disease involving key processes such as invasion, migration and epithelial-to-mesenchymal transition. Numerous studies have shown the key role of miRNAs in the regulation of genes, including transcription factors and signalling pathways related to epithelial cell plasticity, cell invasion, migration and metastasis [30]. The search for biomarkers that contribute to the early identification of these processes is a crucial step in the prevention and treatment of lung cancer patients. Our pilot study demonstrated that the analysis of the expression level of miRNAs in biofluids from the respiratory tract such as EBC has the potential not only to identify the presence and the subtype of lung cancer but also clinical features such as stage, invasion and metastasis.

Most lung cancer patients are diagnosed at late stages and conventional treatments including surgery, chemotherapy and radiotherapy are not effective for those stages, which is translated into a poor survival rate in the short-term. Thus, early diagnosis and accurate prognosis are important factors to improve the survival rate of these patients. There are several cancer-associated miRNAs that can predict the prognosis of lung cancer [31]. A recent meta-analysis carried out by Xiao et al. which included 15 studies and 1753 lung cancer patients in total, identified a signature of 18 miRNAs (8 down and 10 up) from biopsies, serum and plasma associated with poorer overall survival [32]. In the present study, we showed for the first time that the analysis of miRNAs in EBC from lung cancer patients has the capacity to predict the clinical outcome of these patients in non-invasive samples. Thus, the identification and validation of robust miRNA signatures in EBC in large longitudinal studies might constitute a novel valuable tool for the early identification of patients at higher risk.

Interestingly, we also noticed that several miRNAs including miR-1272, miR-6858, miR-5094, miR-6727, miR-320b and miR-548 family were simultaneously associated with several clinical features, such as the presence of the tumor (miR-6858 and miR-5094), type of lung cancer (miR-1272, miR-6858 and miR-6727), metastasis (miR-1272, miR-5094, miR-6727, miR-320b and miR-548 family), invasion (miR-1272 and miR-548 family) and stage (miR-1272, miR-5094, miR-320b and miR-548 family), which reinforces their relevant role as potential biomarkers of this disease.

In addition, we identified potential mRNA targets of the EBC miRNAs known to be related to lung cancer pathogenesis. Thus, individual miRNAs could simultaneously interact with multiples mRNA targets and reciprocally individual mRNA targets could be simultaneously regulated by multiples EBC miRNAs

Among them, KRAS, which is frequently mutated in lung cancer, and EGFR, which is a major therapeutic target in this disease, were also predicted targets of three miRNAs identified in EBC from lung cancer patients.

These results highlighted the likely coordinated dysregulation of the EBC miRNA profile in lung cancer patients which might influence the pathologic mechanism driven by their potential mRNA targets. Future studies are needed to further characterise the relationship between these miRNAs and mRNA targets and to gain insight into the biologic role of miRNAs in EBC.

Limitations of the study: authors recognize the impact of the small sample size may have on the strength of the results, not allowing some kind of analysis such as the effect of the medications or the differentiation among all the stages of the tumor or other clinical features. A larger cohort of patients and healthy donors would be necessary to replicate and validate these results and reach a conclusive model.

In conclusion, our findings demonstrate proof of principle that the integration of unsupervised high-throughput analysis such as the genome-wide miRNA profiling of EBC from lung cancer patients, and advances in computational tools, such as machine learning, could allow the identification, stratification and monitorisation of lung cancer patients with high accuracy in a non-invasive type of sample. This proof of concept paves the way for the development of large clinical studies involving this and other respiratory diseases in the era of personalised medicine that could have a substantial impact on the prognosis of individuals with lung disease.

## Figures and Tables

**Table 1:** Clinical characteristics of lung cancer patients and healthy donors.

	Healthy Donors	Lung Cancer Patients
Number of Subjects	21	21
Gender (men/women)	20/1	19/2
Age (mean ± SD), years	61.33 ± 9.53	62.85 ± 8.06
Smoking (no/yes)	4/17	8/13
Lung Cancer Histology		
Adenocarcinoma (AD)		11
Squamous cell carcinoma (SCC)		10
Stage (I/II/III/IV)		1/3/6/11
Metastasis (no/yes)		10/11

**Table 2:** EBC miRNAs with potential as biomarkers in lung cancer.

Cancer vs. HDs		Type of Cancer: AD vs. SCC		Stage I-III vs. Stage IV	
EBC miRNAs	Combined Model	EBC miRNAs	Combined Model	EBC miRNAs	Combined Model
miR-6777-5p	AUC: 0.837	miR-8075	AUC: 0.981	miR-602	AUC: 0.935
miR-4507	Specificity: 0.75	miR-4529-3p	Specificity: 1	miR-551b-5p	Specificity: 0.88
miR-451a	Sensitivity: 0.81	miR-7704	Sensitivity: 0.88	miR-1272	Sensitivity: 0.91
	**Invasion vs. No Invasion**		**Prognosis 500 days: Deceased vs. Alive**		
	EBC miRNAs	Combined Model	EBC miRNAs	Combined Model	
	miR-6803-5p	AUC: 1	miR-877-5p	AUC: 1	
	miR-548x-3p	Specificity: 1	miR-6777-5p	Specificity: 1	
	miR-1272	Sensitivity: 1	miR-6780a-5p	Sensitivity: 1	

## Data Availability

The datasets used and/or analyzed during the current study are available from the corresponding authors on reasonable request.
